# Chiglitazar Preferentially Regulates Gene Expression via Configuration-Restricted Binding and Phosphorylation Inhibition of PPAR*γ*

**DOI:** 10.1155/2017/4313561

**Published:** 2017-09-19

**Authors:** De-Si Pan, Wei Wang, Nan-Song Liu, Qian-Jiao Yang, Kun Zhang, Jing-Zhong Zhu, Song Shan, Zhi-Bin Li, Zhi-Qiang Ning, Laiqiang Huang, Xian-Ping Lu

**Affiliations:** ^1^Shenzhen Chipscreen Biosciences Ltd., Shenzhen, Guangdong 518057, China; ^2^Shenzhen Key Lab of Gene & Antibody Therapy, Division of Life & Health Sciences, Graduate School at Shenzhen, Tsinghua University, Shenzhen, Guangdong 518057, China

## Abstract

Type 2 diabetes mellitus is often treated with insulin-sensitizing drugs called thiazolidinediones (TZD), which improve insulin resistance and glycemic control. Despite their effectiveness in treating diabetes, these drugs provide little protection from eminent cardiovascular disease associated with diabetes. Here we demonstrate how chiglitazar, a configuration-restricted non-TZD peroxisome proliferator-activated receptor (PPAR) pan agonist with moderate transcription activity, preferentially regulates* ANGPTL4* and* PDK4*, which are involved in glucose and lipid metabolism. CDK5-mediated phosphorylation at serine 273 (S273) is a unique regulatory mechanism reserved for PPAR*γ*, and this event is linked to insulin resistance in type 2 diabetes mellitus. Our data demonstrates that chiglitazar modulates gene expression differently from two TZDs, rosiglitazone and pioglitazone, via its configuration-restricted binding and phosphorylation inhibition of PPAR*γ*. Chiglitazar induced significantly greater expression of* ANGPTL4* and* PDK4* than rosiglitazone and pioglitazone in different cell models. These increased expressions were dependent on the phosphorylation status of PPAR*γ* at S273. Furthermore, ChIP and AlphaScreen assays showed that phosphorylation at S273 inhibited promoter binding and cofactor recruitment by PPAR*γ*. Based on these results, activities from pan agonist chiglitazar can be an effective part of a long-term therapeutic strategy for treating type 2 diabetes in a more balanced action among its targeted organs.

## 1. Introduction

Metabolic syndromes, including type 2 diabetes mellitus (T2DM) and its associated complications (e.g., obesity, cardiovascular symptoms, and dyslipidemia), have significant worldwide impact. Current antidiabetic treatments, especially the insulin-sensitizing class of drugs called thiazolidinediones (TZD) (i.e., rosiglitazone (Ros) and pioglitazone (Pio)), improve insulin resistance and glycemic control with benefit of improvement in rental complication. However, they offer ambiguous protection from eminent cardiovascular risks associated with the diseases. Moreover, the related side effects, such as water retention and body weight gain, rather impair their extended uses in long-term management of diabetic patients. Nevertheless, the TZD class of peroxisome proliferator-activated receptor *γ* (PPAR*γ*) agonists is one major and important therapeutic that directly targets insulin resistance by protecting pancreatic *β*-islet cells, dysregulated transcription program on glucolipid modulation, and imparting anti-inflammatory protection. These drugs also exhibit a more preventive effect and provide more durable HbA1c control than other diabetes treatments [1, 2]. In China, less than one-third of T2D patients received a durable control of HbA1c and only 5.6% of T2D patients with dysregulated metabolic function achieved a comprehensive control of glucose, lipid, and blood pressure [3]. Therefore, development of new type of insulin sensitizers for treating type 2 diabetes and associated complications remains of great interest and potential.

PPAR*γ*, a primary target of TZDs along with PPAR*α* and PPAR*δ*, is a ligand-activated member of the nuclear hormone receptor superfamily which is expressed in various tissues with overlapping or distinct biological activity in controlling glucose, lipid, and energy homeostasis. In brief, PPAR*γ* is predominantly present in adipose tissue and macrophages and functions to repartition fatty acids (FAs) to adipose tissue from muscle, liver, and circulation, thus improving insulin resistance [4]. PPAR*α* is mainly expressed in liver, heart, muscle, and kidney, where it stimulates FA oxidation and improves lipoprotein metabolism [5]. Although PPAR*δ* is expressed ubiquitously, its function is less defined and PPAR*δ* is mainly considered as an important regulator of FA metabolism and thermogenesis [6]. All three subtypes also exert pleiotropic anti-inflammatory effects via distinct mechanisms in modulating proliferation or cholesterol turnover in vascular endothelial cells and macrophages [7]. Controversially, PPAR*α* and PPAR*δ* promote osteoblast activity in bone, while PPAR*γ* promotes osteoclast activity [8]. Considering their mutually compensatory and sometimes antagonistic effects, a less potent and well-balanced agonist that targets all three PPAR subtypes may provide more comprehensive protection from metabolic dysfunction and accompanied cardiovascular disease, as well as offset of undesired side effect such as weight gain and bone fracture associated with highly active PPAR*γ* agonists in T2DM patients [9, 10]. Thus far, no clinically proven drugs have been developed by such strategies, most likely due to difficulty in discriminating one effect from another for the same target or in bringing well-balanced activities from different subtypes.

Recently, several studies reported that specific inhibition of PPAR*γ* phosphorylation, induced by “pathological stimuli” such as a high-fat diet, obesity, and inflammation, at serine 273 (S273) by TZD and synthetic ligands, resulted in alterations in gene expression profile which led to insulin resistance in animals and humans. Therefore, this event may play a unique role in discriminating insulin-sensitizing from other adverse effects, such as weight gain and edema, used to be linked to PPAR*γ* activation by TZDs [11–14].

Chiglitazar (Chi) is a configuration-restricted non-TZD PPAR pan agonist, with AC50 of 1.2, 0.08, and 1.7 *μ*M in CV-1 cells for PPAR*α*, PPAR*γ*, and PPAR*δ*, respectively, which is currently in phase III clinical development in China. This compound is a moderate transcriptional activator of all three PPAR subtypes and induces different patterns of gene regulation compared to the TZD class of drugs* in vitro* and* in vivo* [15, 16]. In animal studies, compared with Ros, Chi demonstrated comparable antidiabetic effects but with fewer adverse effects on body weight and fat pad weight increases in* KKAy* and* db/db* diabetic mouse models [16]. This compound also produced a much improved lipid profile in monosodium L-glutamate- (MSG-) induced obese rats [15].

In this article, we report that Chi preferentially induced two important PPAR target genes,* ANGPTL4* (angiopoietin-like 4) and* PDK4* (pyruvate dehydrogenase kinase, isozyme 4), which are involved in lipid metabolism and insulin sensitization, relative to Ros and Pio. These genes are regulated mainly by PPAR*δ* and to a lesser extent by other PPAR subtypes [17, 18]. The differential induction by Chi did not seem to be entirely due to its pan agonist activity. Instead, our results demonstrated that both genes were directly regulated by CDK5- (cell division protein kinase 5-) mediated phosphorylation of PPAR*γ* at S273, which affects cofactor recruitment by the receptor and binding of a PPAR*γ*-containing transcription complex to* PDK4* and* ANGPTL4* promoters. The unique structure of Chi allows its distinct binding and interactions with the receptor, which results in significantly stronger inhibition of site-specific phosphorylation leading to higher induction of* ANGPTL4* and* PDK4* expression. Since the phosphorylation status of PPAR*γ* is directly linked to “pathological stimuli” such as a high-fat diet, obesity, and inflammation commonly seen in T2DM patients, the prominent activity of Chi in this regard in addition to its pan agonist activity can thus act cooperatively to rebalance glucose, FA uptake, and substrate utilization in energy production upon insulin resistance and obesity, which may improve the clinical prognosis of T2DM patients over time.

## 2. Materials and Methods

### 2.1. Chemicals

Chiglitazar was discovered and synthesized by Chipscreen Biosciences Ltd. [14]. Rosiglitazone and pioglitazone were provided by Jiangsu Depei Chemical Co. Ltd. (Jintan, China). Roscovitine, Sutent, U0126, SR1664, and VX680 were purchased from Selleck Chemicals (Houston, TX, USA). All chemicals were supplied with purity qualified.

### 2.2. Cell Lines

Human preadipocyte-visceral (HPA-v) cells were purchased from ScienCell (Carlsbad, CA, USA) and cultured in preadipocyte medium (PAM, ScienCell) containing 10% fetal bovine serum. The human normal liver cell line L-02 was purchased from the Shanghai cell bank of the Chinese Academy of Sciences (Shanghai, China) and cultured in RPMI-1640 medium containing 10% fetal bovine serum, 50 *μ*g/ml streptomycin, and 50 units/ml penicillin at 37°C in a humidified incubator with 5% CO_2_.

### 2.3. Molecular Docking Simulation (MDS)

The published crystal structures of PPAR*γ* binding to its ligands were downloaded from the Protein Data Bank (PDB code: 2PRG, 2XKW) [19]. Images of structures were generated using UCSF Chimera [20]. MDS was performed using the Molegro Virtual Docker (MVD) [21]. The conformation with the lowest docked energy produced by Chiglitazar against either 2PRG or 2XKW was chosen as the proposed mode. The MDS modes for rosiglitazone and pioglitazone were referenced from published sources [18, 19]. The output structure figures were prepared with PyMOL (DeLano Scientific LLC) [22].

### 2.4. Gene Expression Analysis by Real-Time RT-PCR and Immunoblot

HPA-v cells were cultured overnight in 6-well plates with the appropriate medium before use. After a 24-hour incubation with the tested compounds at varying concentrations or vehicle control (0.1%* vol/vol* dimethyl sulfoxide (DMSO)), cells were collected and total RNA was extracted using TRIzol reagent (Invitrogen, Life Technologies, Carlsbad, CA, USA) followed by purification using the RNeasy Mini Kit (Qiagen, Venlo, Germany, USA). The concentration of RNA was assessed using the UV spectrometer DU520 (Beckman Coulter, Brea, CA, USA). First-strand cDNA was synthesized by SuperScript II reverse transcriptase (Invitrogen) according to the manufacturer's instructions. Real-time PCR was performed with StepOnePlus™ (Applied Biosystems, Life Technologies, Carlsbad, CA, USA) using the FastStart High Fidelity PCR kit (Roche, Molecular Biochemicals, Indianapolis, IN, USA). Primer sequences used were as follows:* CD36,* F: 5′ CATCGCTGGGGCTGTCATT 3′, R: 5′ GCGTCCTGGGTTACATTTTCC 3′;* ANGPTL4,* F: 5′ GCAGCCATTCCAACCTCAA 3′, R: 5′ CAAGAGTCACCGTCTTTCGTG 3′;* PDK4,* F: 5′ ATGTCATTGGCAAGAGGAAGAA 3′, R: 5′ ATTACCAGAAGCACCACAACACT 3′;* LIPE* (lipase, hormone-sensitive), F: 5′ CCCTGCTCCTCCGAGACTT 3′, R: 5′ GGACTTGCGCCCACTTAACT 3′; *β*-actin, F: 5′ AGTTGCGTTACACCCTTTC 3′, R: 5′ TGTCACCTTCACCGTTCC 3′. The relative gene expression level was normalized against *β*-actin. The fold change of expression induced by the various treatments was calculated as the relative expression level in treated samples divided by the vehicle sample or treatment control.

HPA-v cells were cultured in 60 mm plates overnight and then incubated with the indicated compounds at varying concentrations or vehicle control (0.1%* (vol/vol)* DMSO) for 48 hours. Proteins were extracted using the NE-PER Nuclear and Cytoplasmic Extraction Kit and quantitated with Pierce Micro BCA Protein Assay kit according to the manufacturer's instructions (Thermo Fisher Scientific, Waltham, MA, USA). The appropriate quantity of protein was loaded and separated by sodium dodecyl sulfate-polyacrylamide gel electrophoresis (SDS-PAGE) and then transferred to polyvinylidene difluoride membrane (Amersham Biosciences, GE Healthcare Life Sciences, Piscataway, NJ, USA). Blots were blocked for 1 hour at room temperature in TBST buffer (Tris-buffered saline-Tween 20) with 5% nonfat dry milk and subsequently incubated with individual primary antibodies. Primary antibodies for PPAR*γ* (Cell Signaling Technologies (CST), Danvers, MA; 81B8), phospho-(Ser) CDKs substrates (CST; 2324), *β*-Actin (Santa Cruz Biotechnology, San Jose, CA; 69879), LIPE (Abcam, Cambridge, UK; ab45422), CD36 (Abcam; ab17044), PDK4 (Abcam; ab71240), and ANGPTL4 (Abcam; ab95194) were used according to the manufacturer's recommendation. After three washes with TBST buffer, membranes were incubated at room temperature for 1 hour with the respective secondary antibodies according to the manufacturer's recommendation. The bands were visualized with Super ECL Plus Detection Reagent (Applygen, China) and exposed to Kodak X-OMAT film. The film was scanned and transformed to gray-scale graphs with the Bio-Print gel scanner and accessory software (Vilber Lourmat, France).

### 2.5. Cell Transfection for Reporter Gene Assay and Gene Expression Analysis

Cell transfection was performed as described previously using the same constructs for the luciferase reporter assay [12, 13]. Briefly, L-02 cells were seeded into 96-well plates the day before transfection to obtain 50–80% confluence at the time of transfection. For the reporter assay, plasmids expressing hRXR (10 ng), pGFP (10 ng), the relevant PPAR isoform (10 ng), and the corresponding reporter plasmid (30 ng) were cotransfected using FuGENE 6 transfection reagent (Roche) according to the manufacturer's instructions. At 48 hours after transfection, cells were treated with the indicated compounds at various concentrations or vehicle control (0.1%* (vol/vol)* DMSO) for 24 hours. Luciferase activity was measured using a luciferase assay kit from Promega (Madison, WI, USA). GFP fluorescence and luciferase activity were sequentially detected using the Fluoroskan Ascent FL reader (Thermo Labsystems, Helsinki, Finland). Reporter gene expression as represented by luciferase activity was normalized against GFP fluorescence in the same well. Reporter induction by the tested compounds was compared with the vehicle control (i.e., DMSO).

To detect changes in gene expression, L-02 cells were seeded into 6-well plates and cotransfected with plasmids expressing hRXR and different PPAR subtypes. At 48 hours after transfection, cells were incubated with the tested compounds at the indicated concentrations or vehicle control (0.1%* (vol/vol)* DMSO) for 24 hours. Total RNA was extracted and then real-time RT-PCR was performed as described above.

### 2.6. Immunoprecipitation (IP) and Immunoblot of PPAR*γ*

HPA-v cells were cultured in 60 mm plates overnight and then incubated with the indicated compounds or DMSO. Cell lysates were dissolved in Pierce IP Lysis Buffer (Thermo Fisher Scientific) and then incubated with anti-PPAR*γ* primary antibody (CST, 81B8) at 4°C overnight prior to mixing with protein G-Sepharose beads (Thermo Fisher Scientific) for 2 hours. The beads were washed three times with lysis buffer, and the immunoprecipitates were recovered and dissolved in SDS loading buffer. Equal amounts of immunoprecipitates were loaded and separated by SDS-PAGE prior to immunoblot analysis using primary antibodies against PPAR*γ* (CST; 81B8) and phospho-(Ser) CDKs substrates (CST; 2324).

### 2.7. Site-Directed Mutagenesis

Site-directed mutagenesis of amino acid sites in human PPAR*γ*2 (serine 289, glutamine 343, and tyrosine 473) [23] was conducted by inserting full-length human* PPARγ2* into the pcDNA3.1 vector using the QuikChange site-directed mutagenesis kit (Stratagene, La Jolla, CA, USA) as recommended by the manufacturer. The following primer pairs were used for site-directed mutagenesis: PPAR*γ* S289A, F: 5′ GCTGCCAGTTTCGC***G***CCGTGGAGGCTGT 3′, R: 5′ ACAGCCTCCACGGCGCGAAACTGGCAGC 3′, where TCC (serine) was substituted with GCC (Alanine); Y473D, F: 5′ CTCCTGCAGGAGATC***G***ACAAGGACTTGTACTAG 3′, R: 5′ CTAGTACAAGTCCTTGTCGATCTCCTGCAGGAG 3′, where TAC (tyrosine) was substituted with GAC (aspartic acid); E343A, F: 5′ GGGTTCTCATATCCG***C***GGGCCAAGGCTTCA 3′, R: 5′ TGAAGCCTTGGCCCGCGGATATGAGAACCC 3′, where GAG (glutamate acid) was substituted with GCG (alanine). The primer pair and its endonuclease site for subcloning were as follows: NheI F: 5′ CTGGCTAGCGTTATGGGTGAAACTCTGG 3′, XhoI R: 5′ GGCCTCGAGCTAGTACAAGTCCTTGTA 3′. All constructs were confirmed by full-length sequencing.

### 2.8. Chromatin Immunoprecipitation (ChIP)

The Chromatin Immunoprecipitation (ChIP) assay kit (Upstate/Millipore, Temecula, CA, USA) was used according to the manufacturer's instructions. Briefly, after incubation with the indicated treatments, HPA-v cells were fixed with 1% formaldehyde at 37°C for 15 minutes and then sonicated using the XO-900D Ultrasonic cell disruption apparatus (Nanjing, China). After removing debris with protein A-agarose beads, an aliquot of supernatant was taken as the input control. The same sample volume was subjected to immunoprecipitation with anti-PPAR*γ* antibody (CST; 81B8) as described above. The DNA fragments were subsequently recovered using a QIAquick column (Qiagen). Real-time PCR was performed as described above using the following sequence-specific primer pairs against target gene promoters:* ANGPTL4,* F: 5′ TGAGCTCTTCTCCGTTCATCTCGAACCAC 3′, R: 5′ GAGTCTAGACATCTCAGAGGCTCTGCCTG 3′;* PDK4,* F: 5′ GGATTTCAACAGCCAGTGCT 3′, R: 5′ ATAGTGCTGCCCAGTGTGTG 3′;* CD36,* F: 5′ ATTTGTGGTTGGTTGCCAAG 3′, R: 5′ AGGTGATGGGTCTTCACCAG 3′;* LIPE,* F: 5′ CAAGTGATTGGGATGAAGCA 3′, R: 5′ CTAGCCAGCCCAGTCTTCAG 3′; insulin, F: 5′ CTTCAGCCCAGTTGACCAAT 3′, R: 5′ AGGGAGGAGGAAAGCAGAAC 3′. Real-time PCR of the* insulin* promoter was used as a negative control. The respective input samples were taken as internal (loading) controls. Real-time PCR was performed as described above. Promoter binding was evaluated by normalizing the Ct (threshold cycle of PCR) value of samples immunoprecipitated with anti-PPAR*γ* antibody against the Ct of respective Input controls.

### 2.9. *In Vitro* Phosphorylation and the Cofactor Recruitment Assay

Active heterodimer of CDK5/p25 was purchased from Millipore (cat. number 14-516). Recombinant human His-PPAR*γ*-LBD fragment was expressed and purified as described previously [24].* In vitro* phosphorylation of PPAR*γ*-LBD (ligand binding domain) was performed in a 50 *μ*l reaction volume consisting of the individual tested agonists or vehicle control (DMSO) at the indicated concentration, 1 *μ*g of His-PPAR*γ*-LBD, 30–50 ng of active CDK5/p25, and 100 mM ATP in 1x kinase buffer. The reaction was incubated at 30°C for 30 minutes and then subjected to immunoblot analysis or the AlphaScreen assay of cofactor binding.

The representative LXXLL peptide motifs of different cofactors were synthesized according to published literature. The binding of various peptide motifs to PPAR*γ*-LBD was determined using the AlphaScreen assay (AlphaScreen® Histidine (Nickel Chelate) Detection Kit, Perkin-Elmer, Waltham, MA, USA) as described previously [24]. The conventional assay was conducted with approximately 40 nM His-tagged PPAR*γ*-LBD and 40 nM of the respective biotinylated peptides in the presence of 5 *μ*g/ml donor and acceptor beads in a buffer containing 50 nM MOPS, 50 mM NaF, 50 mM CHAPS, and 0.1 mg/ml bovine serum albumin, pH 7.4. For the cofactor binding assay with phosphorylated PPAR*γ*-LBD,* in vitro* phosphorylation was first performed as described above followed by the conventional AlphaScreen procedure. The phosphorylation status of PPAR*γ*-LBD was varied depending on the presence of CDK5/p25, ATP, or tested agonists or their order of addition into the* in vitro* phosphorylation reaction. For example, “LBD + CDK5 − ATP + agonist” represents no ATP in the phosphorylation reaction; “LBD + CDK5 + ATP + agonist” represents that all components were present in the phosphorylation reaction. Furthermore, “(LBD + CDK5 + ATP) + agonist” denotes that* in vitro* phosphorylation was completed before agonist was added. The representative biotinylated peptide motifs used were as follows: NCOR2 (nuclear receptor corepressor 2): Biotin-GHSFADPASNLGLEDIIRKALMGSF; PGC1a (peroxisome proliferator-activated receptor gamma, coactivator 1 alpha): Biotin-QEAEEPSLLKKLLLAPANTQ; SRC1-2 (nuclear receptor coactivator 1): Biotin-SPSSHSSLTERHKILHRLLQEGSP; SRC2-3 (nuclear receptor coactivator 2): Biotin-SPKKKENALLRYLLDKDDT; SRC3-3 (nuclear receptor coactivator 3): Biotin-SPKKKENNALLRYLLDRDD; TRAP1b (mediator complex subunit 1, MED1): Biotin-FSKVSQNPILTSLLQITGN.

The fold change of baseline cofactor recruitment induction is represented as the ratio of the relative luciferase unit (RLU) value from the ligand-free binding reaction with cofactor divided by the value with no peptide in presence. The fold change of cofactor recruitment induction by the tested agonists was calculated as the ratio of the RLU value from the binding reaction with agonist divided by vehicle control (DMSO).

### 2.10. Statistical Analysis

Student's *t*-test was performed to determine statistical significance as needed. Statistical significance between the different comparisons was defined as *p* < 0.01.

## 3. Results

### 3.1. Differential Induction of* ANGPTL4* and* PDK4* Expression by Chiglitazar and TZD Class Compounds

**Figure 1:**
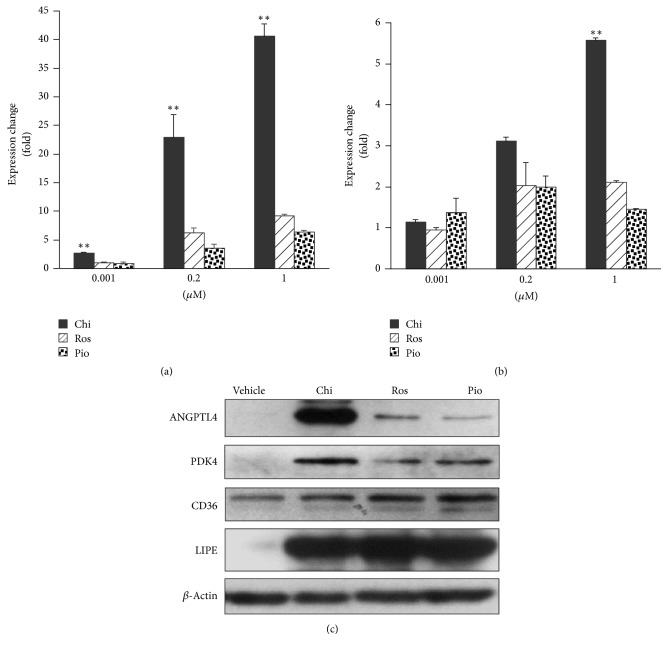
Preferential induction of* ANGPTL4* and* PDK4* mRNA and protein by chiglitazar. (a) and (b) Human preadipocyte HPA-v cells were incubated with different concentrations of the three agonists for 24 hours before total RNA was collected and purified. Real-time RT-PCR was performed as described in* Materials and Methods*. The dose-dependent changes in* ANGPTL4* (a) and* PDK4* (b) expression induced by the different agonists were normalized against the sample treated with vehicle (0.01%* (vol/vol)* DMSO) only. (c) HPA-v cells were incubated with 1 *μ*M of each agonist for 48 hours. Protein levels were assessed by immunoblot analysis using the respective primary antibodies (*β*-Actin, 1 : 1000; LIPE, 1 : 1000; CD36, 1 : 1000, PDK4, 1 : 500; ANGPTL4, 1 : 500) as described in* Materials and Methods*. Chi: chiglitazar; Ros: rosiglitazone; Pio: pioglitazone; LIPE: lipase, hormone-sensitive (HSL). Data represent the average and standard deviation of three independent experiments. *∗∗* represents statistically significant differences in expression induction by Chi relative to Ros and Pio (*p* < 0.01).

During the process of investigating the differential effects of Chi and the TZD compounds on the expression of genes involved in lipid metabolism and insulin sensitization, we identified two genes among multiple known PPAR*γ* targets (Supplementary Figure 1 in Supplementary Material available online at https://doi.org/10.1155/2017/4313561),* ANGPTL4* and* PDK4*, which were preferentially induced by Chi. As shown in Figure 1, in human preadipocyte HPA-v cells, mRNA and protein from* ANGPTL4* and* PDK4* were significantly induced by Chi compared to Ros or Pio. Consistent with previous findings [16], the expressions of* CD36* and* LIPE*, two well-established PPAR*γ* target genes, were similar following the treatments with each compound (Figure 1(c)).

**Figure 2:**
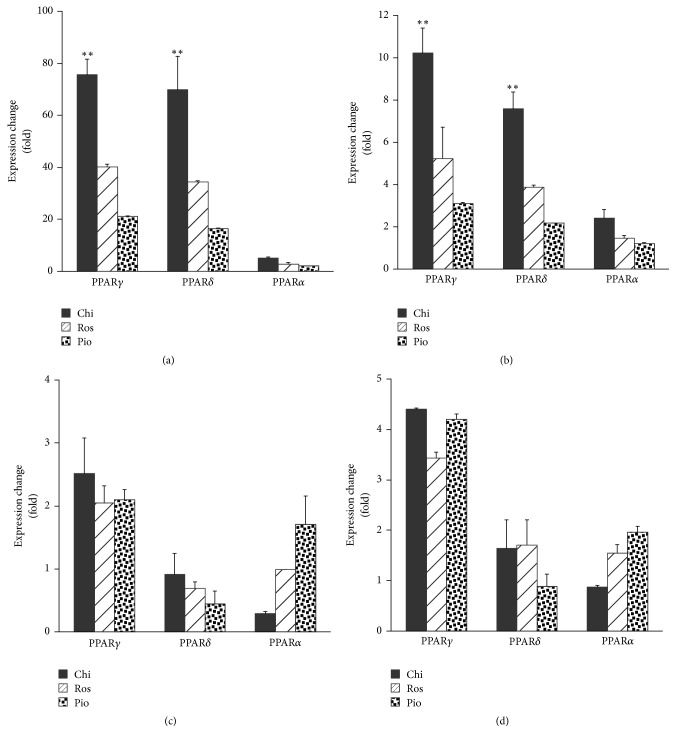
Chiglitazar preferentially induced* ANGPTL4* and* PDK4* expression via both PPAR*γ* and PPAR*δ*. Human hepatic L-02 cells were cotransfected with plasmids expressing the three PPAR subtypes or an empty vector and hRXR. At 48 hours after transfection, cells were incubated with 1 *μ*M of each agonist or the vehicle control (0.1%* (vol/vol)* DMSO) for 24 hours. The mRNA level of each target gene was determined by real-time RT-PCR as described in* Materials and Methods*. Induced expression changes of ANGPTL4 (a), PDK4 (b), CD36 (c), and LIPE (d) were evaluated by comparison against the vehicle control. Data represent the average and standard deviation of three independent experiments. *∗∗* represents statistically significant differences in expression induction by Chi relative to Ros and Pio (*p* < 0.01).

*ANGPTL4* and* PDK4* are regulated by PPAR*δ* and to a lesser extent by other PPAR subtypes [17, 18]. Is the PPAR*δ* activity from pan agonist Chi the only factor that contributes to the observed significantly higher induction of these two genes? We treated a human hepatocyte cell line, L-02, which expresses little endogenous PPARs and their target genes, including* ANGPTL4* and* PDK4*, when treating the cells with Chi, Ros, and Pio (Supplementary Figure 2). As shown in Figure 2, while Chi induced* ANGPTL4* and* PDK4* significantly in PPAR*δ*-transfected cells, this PPAR pan agonist also upregulated both genes even more significantly in PPAR*γ*- but not PPAR*α*-transfected cells compared to Ros and Pio in this cell model. These results suggest that* ANGPTL4* and* PDK4* expressions are upregulated not only in response to PPAR*δ* activation but also by PPAR*γ*. However, this induction of gene expression following agonist treatments does not appear to be correlated with PPAR*γ* transactivity, since Chi consistently produces less potent PPAR*γ* transactivation relative to Ros in different cell model systems [16]. Consistent with this, reporter assays demonstrate that treatment of L-02 cells with Chi, Ros, and Pio yielded AC_50_ for PPAR*γ* transitivity of 0.120 ± 0.047, 0.035 ± 0.037, and 0.288 ± 0.514 *μ*M, respectively (mean ± SD, Supplementary Figure 3).

### 3.2. Chiglitazar and TZD Class Compounds Differentially Bind to PPAR*γ* and Inhibit CDK5-Mediated Phosphorylation

**Figure 3:**
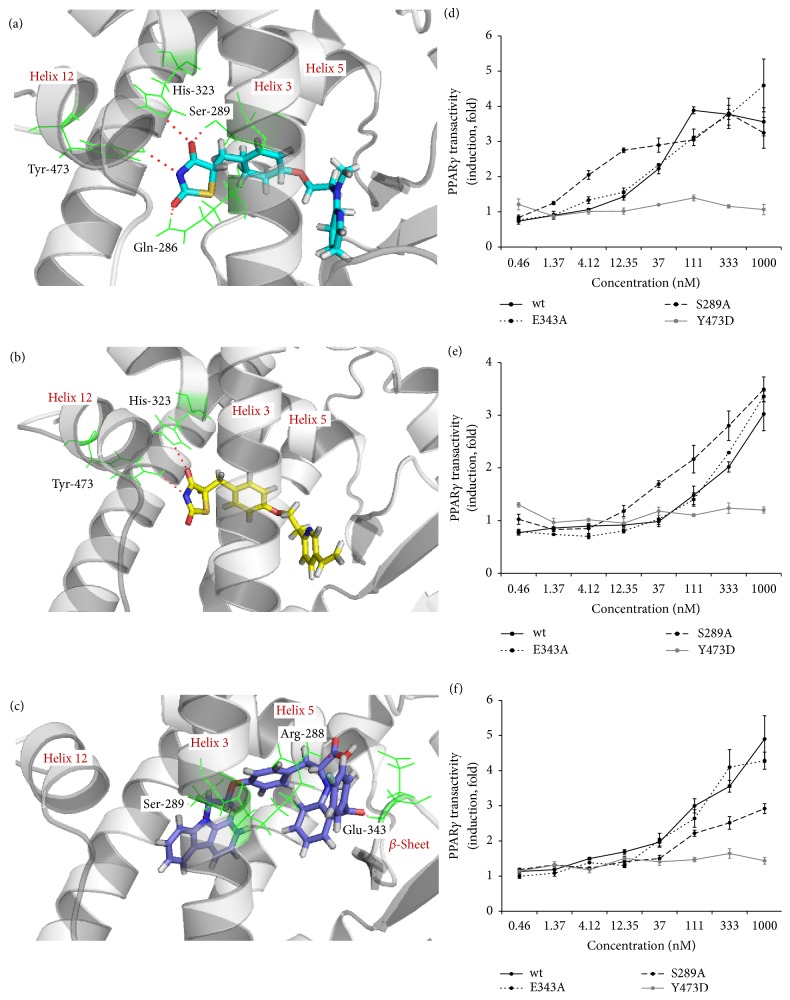
Chiglitazar binds PPAR*γ* differently from rosiglitazone and pioglitazone. (a)–(c) Simulated molecular docking of each agonist to the crystal structure of PPAR*γ* (PDB code: 2PRG, 2XKW as shown in gray) was performed using Molegro Virtual Docker software. Ros ((a), in cyan, docking to 2PRG) and Pio ((b), in yellow, docking to 2XKW) have typical modes of interaction with the receptor via hydrogen bonding to helix 12 (Tyr-473) and helix 5 (His-323) (residues in green, hydrogen bonds shown in dotted red lines). In this conformation, helix 12, along with helices 3–5, forms the coactivator-binding site (AF-2) responsible for full agonist activity. Chi ((c), in blue, docking to 2XKW) does not form typical hydrogen bonding to helices 12 and 5 but rather forms alternative hydrogen bonds to helix 3 and the receptor *β*-sheet (residues in green). This conformation is highly similar to the partial agonist MRL-24. (d)–(g) Site-directed mutation of PPAR*γ* differently affects transactivity of different agonists (Ros (d), Pio (e), and Chi (f)) compared with wild-type receptor by reporter gene assay. PPAR*γ* with site-directed mutation at Y473D, E343A, or S289A, respectively, was constructed and applied in reporter gene assay as described in* Materials and Methods*.

Chi possesses a non-TZD structure with a relatively restricted configuration. Simulated molecular docking with the published PPAR*γ* crystal structure [19] revealed that both Ros (Figure 3(a)) and Pio (Figure 3(b)) form hydrogen bonds with PPAR*γ* via Tyr-473 of helix 12 and His-323 of helix 5, which is consistent with a typical full agonist binding model [25, 26]. However, Chi (Figure 3(c)) did not exhibit any hydrogen bond donor or acceptors in proximity to helices 12 and 5; this compound most likely does not form hydrogen bonds with these two sites. Instead, Chi forms hydrogen bonds with Ser-289 and Arg-288 of helix 3 and Glu-343 of the *β*-sheet. To verify the different binding modes between them, we performed serial site-directed mutations of the referred residues on the receptor including Tyr-473Asp (Y473D), Ser-289Ala (S289A), and Glu-343Ala (E343A), respectively. Unexpectedly, the Y473D mutation significantly diminished the transactivity of Chi as well as Ros and Pio in reporter gene assay (Figures 3(d)–3(f)), despite no hydrogen bond between Chi and PPAR*γ* at this region, which is different from SR1664, a known partial PPAR*γ* agonist, showing no interaction with Y473 (Supplementary Figure 4) [11]. Among other interaction residues, only S289A mutation attenuates the transactivity of Chi which is different from Ros and Pio (Figures 3(d)–3(f)). It seems that Chi might act like a full agonist fashion in terms of ligand-receptor interaction with helices 12 and 5 but differently affect the receptor activity via its configuration-restricted binding mode with particular residues in other regions. Recent studies have shown that PPAR*γ* activity can be modulated by CDK5-mediated phosphorylation at S273 [12], and Glu-343 is adjacent to the reported phosphorylation pocket. Based on this unique receptor-ligand binding model, we hypothesized that, compared with TZDs, Chi might interact with PPAR*γ* by preferentially inhibiting the receptor phosphorylation and, thus, producing a differential pattern of the PPAR*γ*-targeted gene regulation.

**Figure 4:**
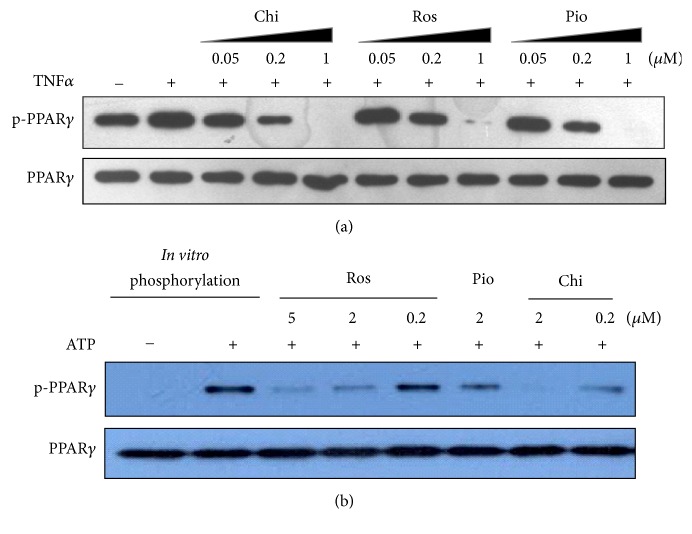
Chiglitazar inhibited TNF*α*-induced and CDK5-mediated phosphorylation of PPAR*γ*. (a) HPA-v cells were cultured in 10 cm plates and incubated with each agonist at the indicated concentration in the presence or absence of TNF*α* (5 ng/ml). Cells were lysed and then subjected to immunoprecipitation (IP) with primary anti-PPAR*γ* antibody followed by SDS-PAGE and immunoblot analysis as described in* Materials and Methods*. (b)* In vitro* phosphorylation was performed using purified active CDK5/p25 (5 ng/mL) and purified recombinant human PPAR*γ*-LBD. Chi, Ros, and Pio were added to the reaction, respectively, at the indicated concentrations in presence of TNF*α* (5 ng/ml) and then incubated for 30 minutes at 30°C. Immunoblot analysis was performed to determine the levels of the specified proteins.

To test this hypothesis, the preadipocyte HPA-v cells were incubated with the ligands in the presence or absence of TNF*α*, an inducer of CDK5-mediated PPAR*γ* phosphorylation [12]. The cell extracts were immunoprecipitated using antibody against PPAR*γ* and then the immunoprecipitates were visualized by immunoblotting using anti-phospho-(Ser) CDKs substrate antibody. While Chi and the two TZDs all exhibited dose-dependent inhibition in TNF*α*-enhanced phosphorylation of PPAR*γ* in HPA-v cells, Chi produced a stronger inhibitory effect even at 0.05 and 0.2 *μ*M (Figure 4(a)). To confirm this result, we performed an* in vitro* phosphorylation reaction with purified recombinant human His-PPAR*γ* ligand binding domain (LBD) [24] and the active heterodimer of CDK5/p25. Our results show that 0.2 and 2 *μ*M Chi inhibits CDK5-mediated phosphorylation of the PPAR*γ*-LBD significantly greater than the two TZDs tested (Figure 4(b)). These experiments demonstrated that Chi exhibited a stronger inhibitory effect on CDK5-mediated PPAR*γ* phosphorylation compared with that of the two TZD compounds.

**Figure 5:**
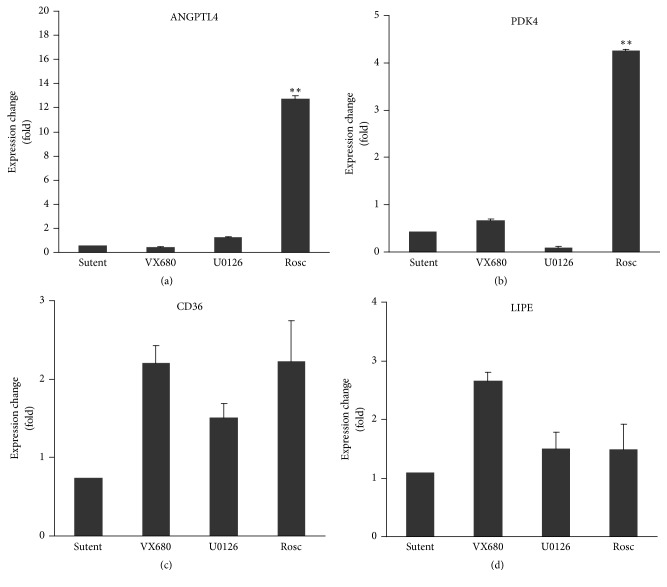
Increased* ANGPTL4* and* PDK4* expressions are associated with inhibition of TNF*α*-induced and CDK5-mediated phosphorylation of PPAR*γ*. HPA-v cells were incubated with different protein kinases (i.e., VEGFR and PDGFR inhibitor Sutent (1 *μ*M), AURK A/B/C inhibitor VX680 (0.1 *μ*M), MEK1/2 inhibitor U0126 (1 *μ*M), and CDK5 inhibitor roscovitine (Rosc, 10 *μ*M) or vehicle control (0.1%* (vol/vol)* DMSO) in the presence of TNF*α* (5 ng/ml) for 24 hours. Total RNA was extracted and subjected to real-time RT-PCR as described in* Materials and Methods*. Data represent the average and standard deviation of three independent experiments. *∗∗* represents statistically significant differences in expression induction by roscovitine compared to all other inhibitors (*p* < 0.01).

To investigate the role of CDK5-mediated PPAR*γ* phosphorylation in regulating target gene expression, we examined PPAR target gene expression in TNF*α*-treated HPA-v cells incubated with a panel of kinase inhibitors (Sutent for VEGFRs and PDGFRs, VX680 for AURK A/B/C, U0126 for MEK1/2, and roscovitine for CDK5). Expressions of* ANGPTL4* and* PDK4* were only induced by roscovitine treatment, indicating that this event is CDK5 kinase-dependent. Meanwhile, induction of the classic PPAR*γ* target genes,* CD36* and* LIPE*, was not changed in HPA-v cells by the treatment with above-mentioned kinase inhibitors (Figure 5), suggesting, at least, that the induction of these two target genes is unlikely due to the phosphorylation status of PPAR*γ*. These results suggest a direct link between CDK5-mediated PPAR*γ* phosphorylation and* ANGPTL4* and* PDK4* expression. Taken together, this differential inhibitory effect of Chi on CDK5-mediated PPAR*γ* phosphorylation perhaps explains why this compound induces* ANGPTL4* and* PDK4* expressions greater than TZDs.

### 3.3. Chiglitazar Differentially Affects Promoter Binding of PPAR*γ*-Containing Transcription Complex and Cofactor Recruitment upon CDK5-Mediated Phosphorylation

**Figure 6:**
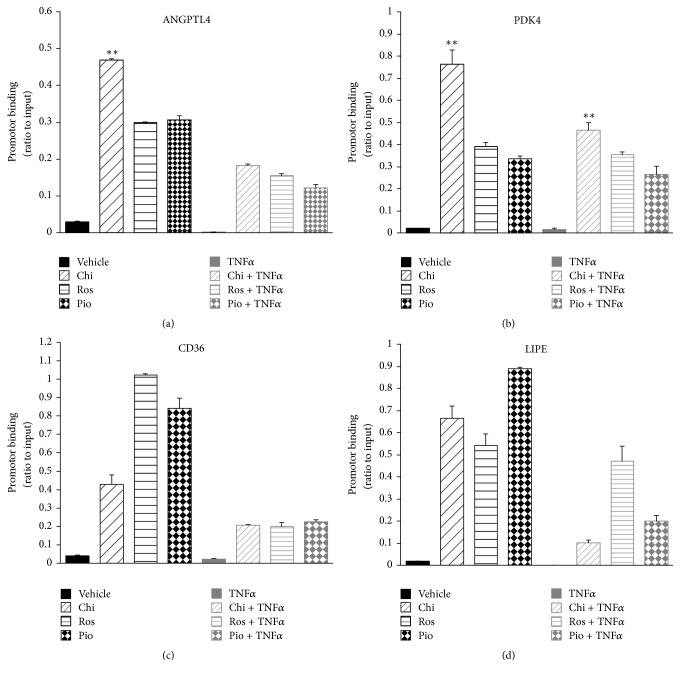
Chiglitazar differentially affects promoter binding of PPAR*γ*-containing transcription complexes. HPA-v cells were incubated with Chi (1 *μ*M), Ros (1 *μ*M), Pio (1 *μ*M), and vehicle control (0.1% (vol/vol) DMSO) in the presence or absence of TNF*α* (5 ng/ml) for 24 hours. ChIP assays using anti-PPAR*γ* antibody were then performed as described in* Materials and Methods*. The relative abundance of promoters bound to PPAR*γ* was normalized against the input control sample. The diagrammed results represent the average of two independent experiments. *∗∗* represents statistically significant differences in promoter binding induced by chiglitazar compared to the other two TZDs (*p* < 0.01).

PPARs heterodimerize with the retinoid X receptor (RXR) to recruit cofactors (e.g., coactivators or corepressors) upon specific ligand binding or to induce protein-protein interactions (e.g., with chromatin-binding proteins) that result in formation of a transcriptome that regulates downstream target genes [27, 28]. Studies have reported that the unique *β*-sheet region of the PPAR*γ*-LBD, in which S273 is located, interacts with the RXR*α* DNA binding domain (DBD) [29, 30]. Thus, it is highly possible that phosphorylation of PPAR*γ* at S273 may affect its interaction with RXR and subsequent promoter binding. We performed ChIP assays to test the promoter binding of PPAR*γ*-containing transcription complex induced by Chi, Ros, and Pio in the presence and absence of S273 phosphorylation. In absence of TNF*α*, it is clear that Chi induced greater recruitment of PPAR*γ*-containing complexes to the* ANGPTL4* and* PDK4* promoters than* CD36* and* LIPE* (Figure 6). Most strikingly, TNF*α* stimulation repressed the binding of PPAR*γ*-containing complexes to all four target gene promoters examined in HPA-v cells treated with the agonists, while Chi seems to keep stronger recruitment of PPAR*γ*-containing complexes to the* ANGPTL4* and* PDK4* promoters compared with the* CD36* and* LIPE* promoters (Figure 6). These results suggest a general, rather than gene-specific, repression of promoter binding by PPAR*γ* in response to TNF*α* via S273 phosphorylation and possibly ERK signaling [31].

**Figure 7:**
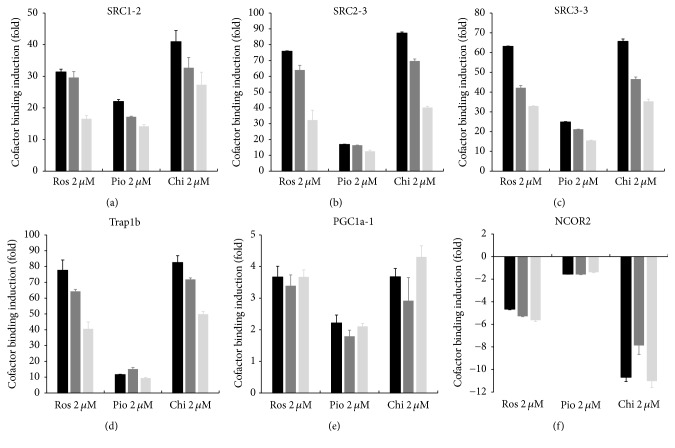
Chiglitazar differentially affects cofactor recruitment upon CDK5-mediated phosphorylation. AlphaScreen assay was performed to evaluate the recruitment of six representative LXXLL peptides to PPAR*γ*-LBD following treatment with 2 *μ*M of Chi, Ros, and Pio as described in* Materials and Methods*. The S273 phosphorylation status of PPAR*γ*-LBD was varied by adding different components to the* in vitro* phosphorylation reaction as indicated. For instance, “LBD + CDK5 − ATP + agonist” denotes no phosphorylation completed by active CDK5/p25 without ATP in the reaction (indicated as black bar). “LBD + CDK5 + ATP + agonist” indicates that the phosphorylation was partially repressed in the presence of agonist (indicated as grey bar). Finally, “(LBD + CDK5 + ATP) + agonist” signifies that the phosphorylation was completed before the addition of agonist to the reaction (indicated as light grey bar). All reactions were then directly applied to the AlphaScreen assay following a conventional procedure as described in* Materials and Methods*. The baseline for cofactor recruitment, independent of ligand binding, was calculated using RLUs from reactions performed in the presence of cofactor peptides divided by control reactions lacking peptide (background signal). Induction of cofactor recruitment by agonist is represented in fold change using RLUs from the cofactor binding reaction with agonist divided by the vehicle control sample (0.1%* (vol/vol)* DMSO).

Next, we performed an AlphaScreen assay using purified recombinant PPAR*γ*-LBD to see whether specific cofactor recruitment is affected by S273 phosphorylation during target gene regulation. Our data show, for the first time, that* in vitro* phosphorylation of PPAR*γ* by CDK5 inhibited cofactor recruitment (Figure 7). Among the six cofactors examined, recruitments of coactivator PGC1a and corepressor NCOR2, which displayed significant baseline binding activity to the LBD independent of ligand binding (Supplementary Figure 5), were not significantly affected by the phosphorylation status of PPAR*γ* at S273. Nevertheless, recruitments of other coactivators, namely, SRC1, SRC2, SRC3, and TRAP1b (MED1), were significantly inhibited by CDK5-mediated phosphorylation of the receptor. The inhibition was partially and differentially restored upon additions of the three ligands, consistent with their inhibition potency on* in vitro* phosphorylation at S273 (Figure 4(b)). NCOR2 served as major corepressor to recruit HDAC (histone deacetylase) to form a complex and further repress transcription activity of PPAR*γ*. Noticeably, there are significantly more chi-bound PPAR*γ*-LBD dissociated from NCOR2 complex than the two TZDs-bound PPAR*γ*-LBD; this differential phenomenon cannot be observed in reporter gene assay.

## 4. Discussion

Although TZD-like drugs such as Ros and Pio are promising therapeutics for the treatment of T2DM patients [1, 2], the potential safety risks linked to cardiovascular diseases and other undesired effects greatly limit their application [32, 33]. New generation of PPAR ligands designated as subtype-selective PPAR modulator, transcriptional-inactive PPAR*γ* synthetic ligand, or pan agonist is currently under preclinical or clinical development [9, 15, 34–38]. The different PPAR subtypes possess overlapping and sometimes antagonistic effects on different aspects of glucose-lipid metabolism, inflammatory regulation, and other biological pathways. The limitations of current knowledge regarding exact mechanism of each subtype and partial inability to further differentiate each other generally halt the development progress [39]. Recent studies have revealed that specific phosphorylation of S273 on PPAR*γ* by obesity-linked or high-fat-diet-induced activation of CDK5, which leads to insulin resistance in animals and humans, can be ameliorated by treatment with TZDs or ligands without transactivation of PPAR*γ* via inhibition of CDK5-mediated S273 phosphorylation [11, 14]. Considering that S273 phosphorylation by CDK5 is unique to PPAR*γ*, this event is important for dictating binding to the promoter of potential target genes related to glucose and lipid regulation, since PPAR*γ* heterodimerizes with DBD region of RXR*α* via the *β*-sheet domain in which S273 resides. Thus, it is essential to understand how the effects of this phosphorylation functionally differentiate PPAR*γ* from other subtypes and the subsequent role of configuration-restricted non-TZD ligands in generating a more balanced or beneficial effect during modulation of glucose/lipid/energy metabolism.

Chi moderately but significantly activates all three PPAR subtypes and the AC_50_ values of them are clinically achievable concentrations. Results from our docking study with the crystal structure of PPAR*γ* demonstrated that Chi does not form a typical binding model as that of TZD-type PPAR*γ* full agonist (e.g., Ros) (Figure 3). Instead, Chi forms hydrogen bonds with Arg-288 of helix H3 and Glu-343 of the *β*-sheet adjacent to the CDK5 phosphorylation pocket. This binding model is very similar to that of MRL-24, a partial agonist of PPAR*γ* [26]. Different agonists binding to the receptor could induce unique conformational changes, as revealed in a recent study utilizing nuclear magnetic resonance, hydrogen/deuterium exchange, and docking [40–42]. Ros has been shown to stabilize the AF2 domain and the *β*-sheet upon binding to the receptor, while MRL-24 mainly stabilizes the *β*-sheet. Despite the lack of cocrystal data for Chi, our docking study and reporter gene activation result with serial site-directed mutations are consistent with published literature showing that stabilization of the *β*-sheet is important in phosphorylation inhibition. As expected, Chi exhibited much stronger inhibition of CDK5-mediated phosphorylation of PPAR*γ* at S273 in HPA-v cells (Figure 4(a)) and* in vitro* phosphorylation assays (Figure 4(b)) than the other two TZDs tested.

The increase in S273 phosphorylation has been linked to insulin resistance in obesity or T2DM patients, probably due to altered expression of genes involved in glucose, lipid, and energy homeostasis [11, 12]. Here, we demonstrated that S273 phosphorylation was directly related to the transcriptional regulation of two insulin resistance-related genes,* ANGPTL4* and* PDK4*. In the human preadipocyte HPA-v cell line, Chi differentially regulated a different set of target genes compared to Ros and Pio. Among them, expression of* ANGPTL4* and* PDK4* was dramatically higher following Chi treatment (Figure 1). This induction was specifically related to TNF*α* stimulation and CDK5-mediated S273 phosphorylation of PPAR*γ*, since the CDK5 kinase inhibitor roscovitine mimicked the effect of Chi (Figure 5). Most strikingly, the expression of* ANGPTL4* and* PDK4* at the gene and protein levels appeared to be preferentially induced by Chi. However, other insulin sensitization-related genes such as* CD36* and* LIPE*, which are apparently not regulated by TNF*α* or CDK5-mediated S273 phosphorylation on PPAR*γ* (Figure 5), displayed comparable expression with all three agonists (Figures 1 and 2). These results suggest a clear distinction between TZD and non-TZD type compounds such as Chi in regulation of insulin resistance, consistent with other reports demonstrating that CDK5-mediated S273 phosphorylation on PPAR*γ* independently regulates a specific set of insulin-sensitizing related genes [11, 12].

Our ChIP and AlphaScreen assays revealed that TNF*α* stimulation and CDK5-mediated phosphorylation indeed inhibit the promoter binding of PPAR*γ*-containing transcription complexes on target genes, as well as cofactor recruitment. Chi could partially restore the recruitment activity of some cofactors (i.e., SRC1, SRC2, SRC3, and TRAP1b) but not others (i.e., NCOR2 and PGC1*α*) inhibited by CDK5-mediated S273 phosphorylation (Figure 7). These data are supported by a previous structural study suggesting that SRC3 interacts with a PPAR*γ* region located far from helix 12 yet near the *β*-sheet containing the CDK5 binding site [11]. Although a previous study indicated that the PPAR*γ* A/B-domain (N-terminal ligand-independent activation function 1, AF-1) was specifically involved in the recruitment or stabilization of cAMP response element-binding protein and p300-containing cofactor complexes to a subset of target genes [43], our understanding of how a particular target gene is regulated by an individual cofactor recruited by PPAR remains poor. We have not identified the differences in recruitment among the six cofactors tested, which may contribute to the increased* ANGPTL4* and* PDK4* expressions promoted by Chi compared with the other two TZDs. In contrast, we found that Chi more significantly dissociates corepressor NCOR2 from PPAR*γ*-LBD than Ros in AlphaScreen assay; knockout NCOR in adipocyte was recently reported to repress PPAR*γ* S273 phosphorylation and increase insulin sensitivity in mouse model [13]; the more potency on dissociation of NCOR2 from PPAR*γ* by Chi partially contributes to its phosphorylation inhibition. In contrast, another report demonstrated that short chain FA butyrate could induce a unique set of PPAR target genes, including* ANGPTL4*, even though no significant activity in cofactor recruitment (except for induced binding of two corepressors NCOR1 and NCOR2 binding) was observed by PamChip® arrays [44]. Butyrate is a HDAC inhibitor, which may be recruited by NCOR2 into PPAR*γ* transcriptome; it is possible to induce* ANGPTL4* expression via interfering in the interaction of HDAC and NCOR2. Considering the pathophysiological and therapeutic relevance of PPAR*γ* S273 phosphorylation in T2DM and its associated complications, the impact of this biological process on specific target gene regulation and cofactor recruitment is worthy of further investigation.

Insulin resistance, T2DM, and its cardiovascular complications reflect a very complex outcome due to dysfunction in the mutually compensatory interactions among glucose, lipid, and energy utilization in relevant tissues. One consequence, namely, lipid overload in adipose tissue, liver, muscle, and heart tissue due to a metabolic syndrome often seen in T2D patients, is a critical factor leading to body weight gain, heart failure, and other cardiovascular conditions. Therefore, treatments that restore glucose, lipid, and energy homeostasis would potentially provide the most clinical benefit. Among the three subtypes, PPAR*α* and PPAR*δ* could contribute additional effects to lipid metabolism over PPAR*γ*. In fact, Pio brings cardiovascular benefits to patients most likely due to weaker PPAR*γ* and PPAR*α* activation compared with Ros [45, 46]. Such a complex mechanism of action for insulin sensitization by PPAR*γ* and other PPAR subtypes may not only explain the drastic differences in clinical outcomes, such as CV events, resulting from the same chemical class of TZDs (e.g., Pio versus Ros) but also potentially enable the development of configuration-restricted non-TZD molecules with fewer side effects associated with PPAR*γ* activation.

Beyond its moderate pan agonist activity, Chi greatly induces two target genes,* ANGPTL4* and* PDK4*, involved in glucose and lipid metabolism via inhibition of PPAR*γ* phosphorylation. Dietary saturated fatty acids (SFAs) can be presented to cells in the form of circulating free fatty acids (FFAs) or FAs hydrolyzed by lipoprotein lipase (LDL) from chylomicrons or VLDL in the lymph nodes and blood. SFAs are strong proinflammatory nutrients that can trigger activation of intracellular inflammatory pathways in innate immune cells such as macrophages, as well as the main insulin target cells, adipocytes, myocytes, and hepatocytes, leading to insulin resistance, obesity, and cardiovascular disease [47, 48].* ANGPTL4* is an endogenous inhibitor of LDL activity and plays a crucial role in preventing fat-induced inflammation elicited by SFAs, diet-induced obesity, and myocardial infarction through preservation of vascular integrity [49–51].* ANGPTL4* decreases circulating FFAs derived from triglyceride- (TG-) rich lipoproteins to enhance insulin sensitivity of target organs. The positive relationship of* ANGPTL4* activity with increased plasma level of TG-rich VLDL lipoproteins is seen in human with less-of-function variance* ANGPTL4* E40K as well as* ANGPTL4*-deficient or liver-specific overexpression transgenic mice [52–54]; however, transgenic overexpression of* ANGPTL4* suppresses foam cell formation to reduce atherosclerosis development in atherosclerosis-prone E3L mice [55]. Similar to lipotoxic cardiomyopathy caused by cardiac-specific overexpression of LPL, heart function is also impaired in transgenic mice with cardiac-specific overexpression of* ANGPTL4* [56], highlighting the importance of balanced FFA demand and availability for normal cardiac function. Thus,* ANGPTL4* may potentially contribute to increased lipid accumulation (i.e., lipid overload) in the liver, muscle, and heart [57], which is a “side effect” that can be overcome by increased *β*-oxidation in these tissues through the additional activation of PPAR*α* and PPAR*δ* activity [58]. Indeed, plasma TG level was increased by Ros but was reduced by Pio in clinical application. It could be partially due to stronger induction of* ANGPTL4* by Ros and a weak PPAR*α* transactivity of Pio [44, 59]. In preclinical studies of various animal models, Chi exhibited different gene expression profiles, including the preferential induction of* UCP-1* in fat and skeletal tissues [15]. Furthermore, no body weight gain in leptin-positive KKAy mice and less fat pad weight increase in leptin-negative* db/db* mice were noted after treatment by Chi [16]. Much less effect on increase in heart weight in long-term toxicity rat study and no increase of heart weight in dog upon Chi treatments were also observed [16].

Increased insulin sensitivity occurs following enhanced FA reesterification in adipose tissue, reduced FA efflux, and limited ectopic lipid deposition.* PDK4* inactivates the pyruvate dehydrogenase complex, which leads to reliance on *β*-oxidization of FFA as a primary energy source rather than glucose. The glycerol-3-phosphate catalyzed from glucose thus serves to immobilize circulating FFA in adipose tissue, which further enhances the insulin sensitivity of other glucose-utilizing organs.* PDK4* is upregulated in skeletal muscle in insulin-resistant states and proposed to be involved in the etiology of insulin resistance [60]. However, systemic* PDK4* knockout in mice has no obvious phenotype change and no effect on blood glucose levels and insulin sensitivity in the fed state and only leads to hypoglycemia after the prolonged starvation [61], which is consistent with the essential role of* PDK4* for glucose homoeostasis. Although cardiac-specific overexpression of* PDK4* exacerbates hypertrophic cardiomyopathy caused by calcineurin stress-activated pathway in* PDK4/CnA* double transgenic mice [62], it exerts a protective effect against cardiac ischemia-reperfusion injury via chronic metabolic adaptation in similar cardiac-specific PDK4 transgenic mice [63]. Myocardiocytes in T2DM patients exert less substrate flexibility for energy production and manifest diabetic cardiomyopathy or later stage of heart failure in absence of other macrovascular complications. In addition to* PDK4*, distinct metabolic modulation profile of PPAR*δ* from two other PPAR subtypes and cardiac protection in ischemia/reperfusion mice as previously reported [64] may endow PPAR pan agonist such as Chi benefit in the prevention of diabetic cardiac dysfunction.

## 5. Conclusions

In summary, we demonstrate that regulation of the expression of* ANGPTL4* and* PDK4*, which are important regulators of glucose and lipid metabolism and insulin resistance, is directly linked to S273 phosphorylation of PPAR*γ* in response to common T2DM factors such as a high-fat diet, obesity, and inflammation. Chi preferentially induced* ANGPTL4* and* PDK4* expressions by inhibiting these cascades most likely via its unique configuration-restricted binding to the receptor, which influenced cofactor recruitment and promoter binding of the PPAR*γ*-containing transcription complex. Further investigation is required to elucidate the detailed mechanism for gene-specific regulation by the phosphorylated receptor. Nevertheless, the effect of Chi on the regulation of insulin resistance-related gene expression by PPAR*γ*, together with its moderate activity on PPAR*α* and *δ*, could rebalance glucose and FA uptake and substrate utilization in adipose tissue, muscle, liver, and heart upon insulin resistance and obesity, which may provide comprehensive clinical benefits to T2DM patients in the future.

## Supplementary Material

Supplementary Figure 1: The expression induction of target genes by three agonists in human pre-adipocyte HPA-v cells. Supplementary Figure 2: The expression induction of target genes by three agonists in L-02 cells. Supplementary Figure 3: PPARγ transactivation of three agonists in L-02 cells. Supplementary Figure 4: The transactivation of SR1664 on different PPARγ constructs in L-02 cells. Supplementary Figure 5: In vitro cofactors recruitment by PPARγ LBD independent of ligand.
