# Supplementary material for: Chiglitazar Preferentially Regulates Gene Expression via Configuration-Restricted Binding and Phosphorylation Inhibition of PPARγ
Source: PPAR Res. 2017 Sep 19;2017:4313561. doi: 10.1155/2017/4313561 (PMC5625810; doi:10.1155/2017/4313561)

**Supplementary Figure 1: The expression induction of target genes by three agonists in human pre-adipocyte HPA-v cells.**

Human pre-adipocyte cells HPV-v were incubated with Chiglitazar (Chi, 1μM), Rosiglitazone (Ros, 1μM), Pioglitazone (Pio, 1μM) or vehicle control (0.1% of DMSO) for 24 hours. The relative expression level of target genes were evaluated with Realtime RT-PCR as described in *Materials and Methods*. The expression change of target genes induced by three agonists were calculated by normalization with internal control gene (β-actin) and comparing to vehicle control.

**Supplementary Figure 2: The expression induction of target genes by three agonists in L-02 cells.**

Human hepatocyte cells L-02 were incubated with Chiglitazar (Chi, 1μM), Rosiglitazone (Ros, 1μM), Pioglitazone (Pio, 1μM) or vehicle control (0.1% of DMSO) for 24 hours. The relative expression level of target genes were evaluated with Realtime RT-PCR as described in *Materials and Methods*. The expression change of target genes induced by three agonists were calculated by normalization with internal control gene (β-actin) and comparing to vehicle control. There were no significant expression induction of all target genes (ANGPTL4, PDK4, CD36 and LIPE) by three agonists in normal L-02 cells.

**Supplementary Figure 3: PPARγ transactivation of three agonists in L-02 cells.**

L-02 cells were co-transfected with human PPARγ and its specific reporter gene as described in *Materials and Method*. 48 h after transfection, cells were treated with three agonists (Chi, chiglitazar; Ros, rosiglitazone; Pio, pioglitazone) at different concentrations or vehicle control (0.1% of DMSO) for 24 h. Cells were lysed and prepared for the measurement of luciferase activity. PPARγ transactivation of tested agonist was represented as the reporter fluorescence induction, by normalization with paralleled transfection control (GFP fluorescence) and then comparing to vehicle control. The AC50 of Chi, Ros and Pio by the reporter assay in L-02 was 0.120±0.047, 0.035±0.037 and 0.288±0.514 μM, respectively.

**Supplementary Figure 4: The transactivation of SR1664 on different PPARγ constructs in L-02 cells.**

L-02 cells were co-transfected with two human PPARγ constructs (wild type or Y473N respectively) and its specific reporter gene as described in *Materials and Method*. 48 h after transfection, cells were treated with SR1664 at different concentrations or vehicle control (0.1% of DMSO) for 24 h. Cells were lysed and prepared for the measurement of luciferase activity. The PPARγ transactivation activity was represented as the reporter fluorescence induction, by normalization with paralleled transfection control (GFP fluorescence) and then comparing to vehicle control.

**Supplementary Figure 5: In vitro cofactors recruitment by PPARγ LBD independent of ligand.**

The purified recombinant human PPARγ-LBD was incubated with 6 different synthesized cofactor peptides respectively and then applied for AlphaScreen assay as described in *Materials and Methods*. The cofactors recruitment was represented as the photon counts in ligand-free binding reaction.

**Supplementary Figure 1**


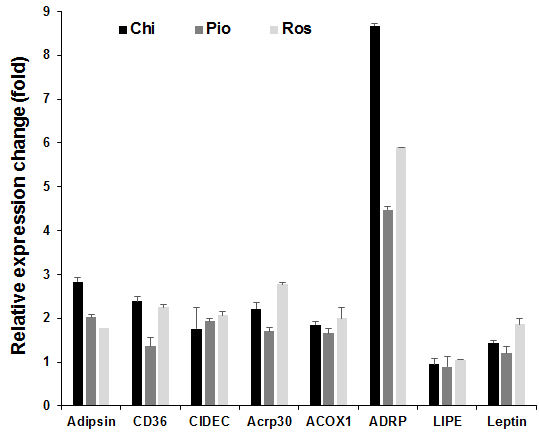


**Supplementary Figure 2**

**Supplementary Figure 3**

**Supplementary Figure 4**


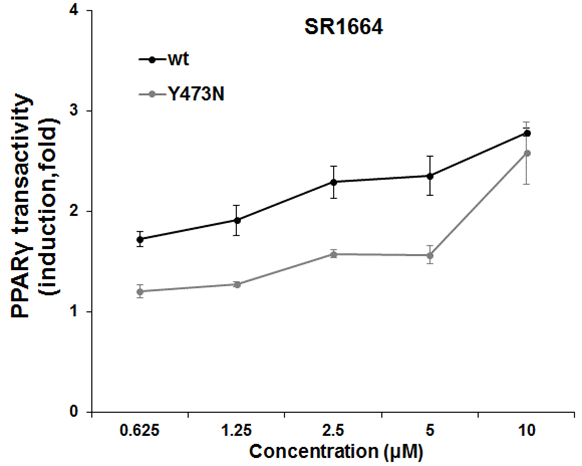


**Supplementary Figure 5**


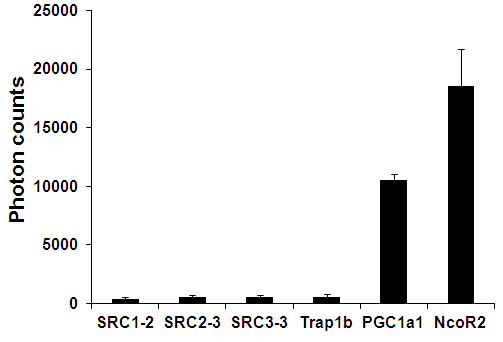

Supplement: Supplementary file 1 — Supplementary Figure 1: The expression induction of target genes by three agonists in human pre-adipocyte HPA-v cells. Supplementary Figure 2: The expression induction of target genes by three agonists in L-02 cells. Supplementary Figure 3: PPARγ transactivation of three agonists in L-02 cells. Supplementary Figure 4: The transactivation of SR1664 on different PPARγ constructs in L-02 cells. Supplementary Figure 5: In vitro cofactors recruitment by PPARγ LBD independent of ligand. [file 4313561.f1.docx]
